# Patterns and patient factors associated with loss to follow-up in the Muhimbili sickle cell cohort, Tanzania

**DOI:** 10.1186/s12913-020-05998-6

**Published:** 2020-12-14

**Authors:** Upendo Masamu, Raphael Z. Sangeda, Daniel Kandonga, Jesca Ondengo, Flora Ndobho, Bruno Mmbando, Siana Nkya, Khadija Msami, Julie Makani

**Affiliations:** 1 Muhimbili University of Healthy and Allied Sciences Sickle Cell Program, Dar es Salaam, Tanzania; 2 Department of Pharmaceutical Microbiology, Muhimbili University of Healthy and Allied Sciences, Dar es Salaam, Tanzania; 3 Muhimbili National Hospital, Dar es Salaam, Tanzania; 4 National Institute for Medical Research, Tanga, Tanzania; 5 Dar es Salaam University College of Education, Dar es Salaam, Tanzania; 6 Department of Hematology and Blood Transfusion, Muhimbili University of Healthy and Allied Sciences, Dar es Salaam, Tanzania

**Keywords:** Muhimbili sickle cell cohort, Loss to follow-up, Sickle cell disease, Sickle cell patient, Muhimbili national hospital, Muhimbili University of health and allied sciences, Tanzania

## Abstract

**Background:**

Monitoring patient’s clinical attendance is a crucial means of improving retention in care and treatment programmes. Sickle cell patients’ outcomes are improved by participation in comprehensive care programmes, but these benefits cannot be achieved when patients are lost from clinical care. In this study, patients are defined as loss to follow-up when they did not attend clinic for more than 9 months. Precise information on the retention rate and characteristics of those who are not following their clinic appointments is needed to enable the implementation of interventions that will be effective in increasing the retention to care.

**Method:**

This was a retrospective study involving sickle cell patients registered in the Muhimbili Sickle Cohort in Tanzania. Descriptive and survival analysis techniques both non-parametric methods (Kaplan-Meier estimator and Log-rank test) and semi-parametric method (Cox’s proportional hazard model), were used. A *p*-value of 0.05 was considered significant to make an inference from the analysis.

**Results:**

A total of 5476 patients were registered in the cohort from 2004 to 2016. Of these, 3350 (58.13%) were actively participating in clinics, while 2126 (41.87%) were inactive, of which 1927 (35.19%) were loss to follow-up. We used data from 2004 to 2014 because between 2015 and 2016, patients were referred to other government hospitals. From the survival analysis results, pediatric (HR: 14.29,95% CI: 11.0071–18.5768, *p* <  0.001) and children between 5 and 17 years [HR:2.61,95% CI:2.2324–3.0705, *p* <  0.001] patients were more likely to be loss to follow-up than the adult (18 and above years) patients. It was found that patients with above averages for hematocrit (HR: 2.38, 95% CI: 1.0076–1.0404, *p* = 0.0039) or mean cell volume (HR: 4.28, (95% CI: 1.0260–1.0598, *p* < 0.001) were more likely to be loss to follow-up than their counterparts.

**Conclusion:**

Loss to follow-up is evident in the cohort of patients in long term comprehensive care. It is, therefore, necessary to design interventions that improve patients’ retention. Suggested solutions include refresher training for health care workers and those responsible for patient follow-up on techniques for retaining patients and comprehensive transition programs to prepare patients who are moving from pediatric to adult clinics.

## Background

Sickle cell disease (SCD) is an inherited disease caused by a single-gene mutation affecting the β-globin gene on chromosome 11. It results in an abnormal hemoglobin protein (HbS) which affects the shape and function of red blood cells (RBC) and subsequently impacts on nearly all organ systems of the body [1]. SCD is one of the most prevalent inherited blood disorders, with an estimated 300,000 individuals born each year with SCD, the highest burden being in Africa where up to 75% of SCD births occur [2]. It is estimated that 50–80% of infants born with SCD in Africa die before the age of 5 years [3]. This disease impacts on both patients and societies, with high morbidity and mortality and reduced quality of life, resulting in increased financial burdens on individuals, families and health care services [1, 4].

Studies have shown that sickle cell clinics have improved the health of sickle cell patients through the provision of comprehensive care. This includes supplying patients with folic acid, treated bed nets, anti-malarial chemoprophylaxis, hydroxyurea for managing pain crises and other services such as health education and genetic counseling [5, 6]. Children under the age of six are provided with daily oral penicillin and pneumococcal conjugate vaccine (PCV), which prevents severe pneumonia episodes, a leading cause of morbidity and mortality in young children with SCD [7]. It has been shown that these services are more effective if patients adhere to their clinical appointments [8].

Loss to follow-up (LTFU), where patients do not attend their clinic appointment (more than 9 months for our study) and cannot be contacted, remains one of the significant challenges facing most clinical cohort studies [9]. When analyzing cohort data with many LTFU events, there is a potential that bias may be introduced into the data. This is if those who are lost differ from those who are retained in the studies and consequently, the effectiveness of treatments may not be correctly assessed [10–12]. Studies on LTFU in relation to HIV have identified several reasons why patients are lost, such as being in good health, financial problems and the clinic being a long way from a patient’s home [9, 13–16] . Most existing studies on SCD are focused on prevalence, hematological profile and hydroxyurea (HU) adherence [17, 18]. There is limited literature on the pattern and factors associated with LTFU. Findings by other authors point that phone calls to remind patients of their clinic appointment are not sufficient to improve patient attendance at the clinic. This was also observed in this cohort in which patients were reminded of their appointment by phone calls and yet missed their clinic appointments. Understanding the patterns and factors associated with those who are LTFU will enable measures to be introduced to reduce LTFU and improve the overall performance of cohort treatment and the patient’s health. The objective of this study is to describe patterns and patients factors associated with LTFU in sickle cell patients registered in Muhimbili Sickle Cohort (MSC) and attended clinics at Muhimbili National Hospital (MNH) from 2004 to 2014.

## Method

### Study design and population

This is a retrospective cohort study using secondary data to describe patterns and patients’ factors associated with LTFU among SCD patients registered in the MSC. The MSC was a descriptive cross-sectional hospital-based study that was established by MNH in collaboration with Muhimbili University of Health and Allied Sciences (MUHAS). The MSC was established to document the clinical spectrum of the disease, identify causes of morbidity and mortality and to develop strategies for appropriate interventions. Between March 2004 and March 2016, a total of 8484 individuals were seen and given unique demographic identity numbers. Testing for SCD was performed using the sickling test. A total of 5476 (64%) individuals were confirmed to have SCD (HbSS), 2121 (25%) with sickle cell trait (HbAS) and 897 (11%) had normal hemoglobin (HbAA). Apart from a few patients with S/β-thalassemia, more than 99% of patients were homozygous for the sickle mutation (HbSS). Individuals who were confirmed to have SCD were enrolled in the cohort.

### Patient management

Demographic data, clinical and laboratory outcomes of every patient registered in the MSC were documented using paper base case report forms (CRF) and in-hospital case files. A convenience sampling method was used with patients identified from the inpatient unit, through screening at entry and during follow-ups. Patients’ information such as date of clinic attended, date of next clinic, age, sex and region of birth were recorded as well as clinical and laboratory results. The programme provided all the diagnoses and medicines in the MSC: the only cost the patient incurred was transport costs to the clinic.

### Study outcome

The definition of LTFU will depend on the schedule of treatment and appointments appropriate for a particular disease. In an HIV study, LTFU was defined as “when a patient has passed three months without having a drug at hand” [19]. MSC patients were referred back to the clinic at an interval of 3 to 6 months; hence in our study LTFU, was defined not attending the clinic for more than 270 days (9 months). Since LTFU classification was done by taking the date difference between two clinic appointments, there is a chance that a patient was LTFU at one point then later returned to care. Hence to avoid that person from being classified as LTFU, we added another criterion that if that patient was not present 9 months before the end of the study, that patient was considered as LTFU.

### Sample population

A total of 5476 patients with confirmed HbSS status were enrolled in the MSC between 2004 and 2016. Three sets of data were collected; i) visit dates and demographic information, ii) clinical results (patient’s routine checks) and iii) test results (hematological parameters). The three datasets were merged based on the unique demographic id, visit id and visit date. Clinical tests were not done at every clinic visit; hence some visits only recorded basic information. As a result, during the data merge, we found missing records that were removed.

### Data analysis

The data were analyzed using R Studio 1.2.5033 [20] and Microsoft Excel (2010). Survival analysis techniques, both non-parametric methods (Kaplan-Meier estimator and Log-rank test) and semi-parametric method (Cox’s proportional hazard model), were used. A *P*-value cutoff of 0.05 was used to confirm the statistical significance of the results. The following analysis assumptions were used in our study. SCD patients who did not attend the clinic for more than 9 months after their previous clinic attendance date were classified as LTFU. Patients were divided into two groups, active patients who were following clinic appointments (did not miss clinic appointment more than 9 months between consecutive clinic appointments) and inactive patients, which included those who are LTFU and those reported dead. Patient total time in the study was taken as time difference between the first and last clinic attendance dates. For the analysis of clinical and laboratory results, we used an event record closest to the last attendance date for the patient. Age was calculated twice, at registration and at the time of the loss to follow-up event. Patients who were reported dead and those who were present in the last nine-month window (a reference to 1/04/2014–30/12/2014) were censored. All survival times were assumed independent and censoring occurred at the right.

## Results

**Fig. 1:**
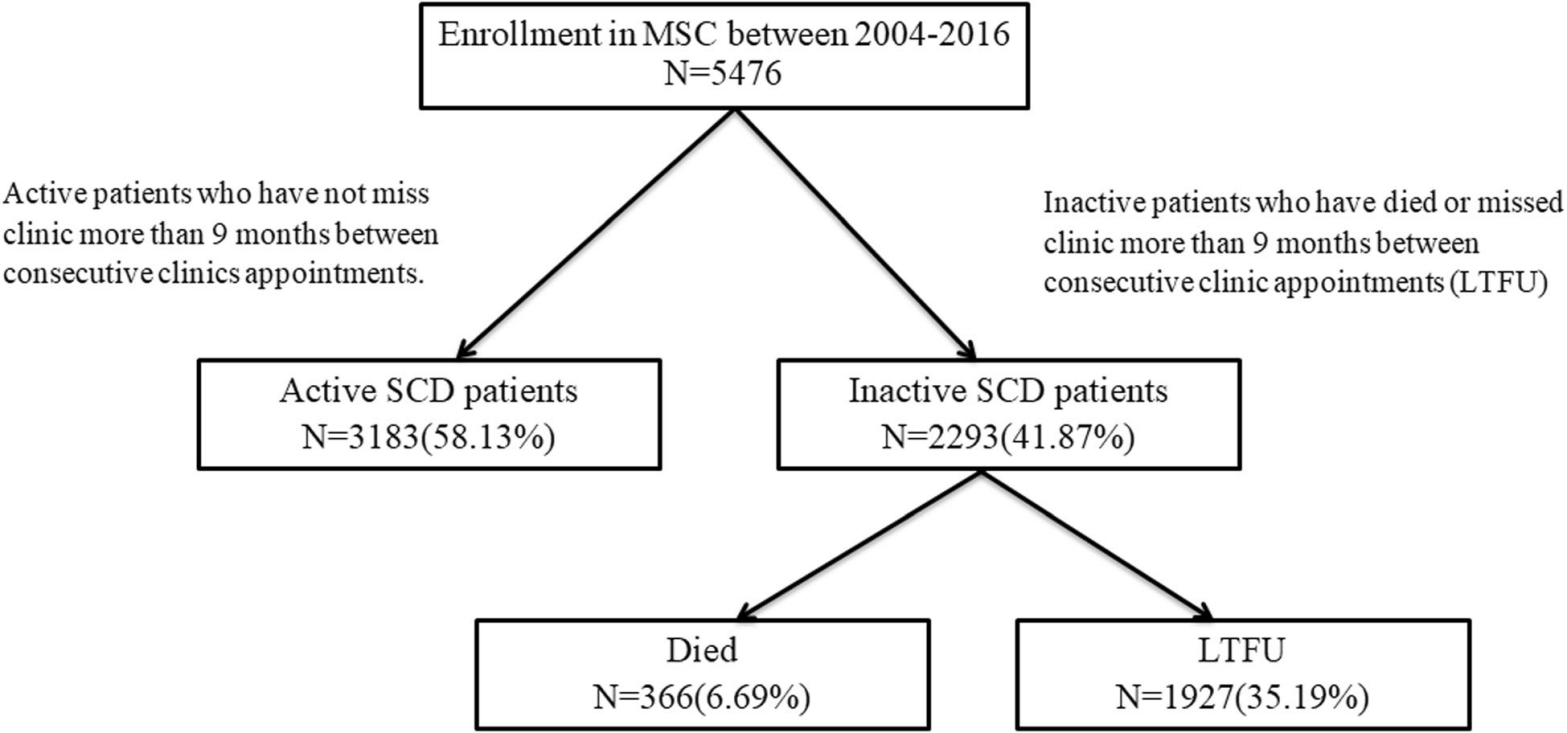
A flow diagram describing the patients enrolled into Muhimbili sickle cohort and their various outcomes

Out of the 5476 registered patients, 3183 (58.13%) were actively following their clinic appointments, while 2293 (41.87%) were inactive on 30/12/2014, 366 of the inactive group were reported dead (Fig. 1). 41.89% were children under 5 years, 45.38% were 5–17 years old while children under 1 year were 402 (7.34%). 2797 (51.08%) were males, while females were 2679 (48.92%) (Table 1). Most of the enrolled patients (72.92%) were born in the Coastal regions such as Dar es Salaam and Tanga.

**Table 1:** Demographics characteristics of patients enrolled in the Muhimbili Sickle Cohort

	Total
All SCD	5476
**Age groups**	
0–4	2294 (41.89%)
5–17	2485 (45.38%)
18 and over	680 (12.42%)
**Gender**	
Female	2679 (48.92%)
Male	2797 (51.08%)
**Place of Birth**	
Coastal Regions	3993(72.92%)
Others	1476 (26.95%)
Missing	7 (0.13%)

**Fig. 2:**
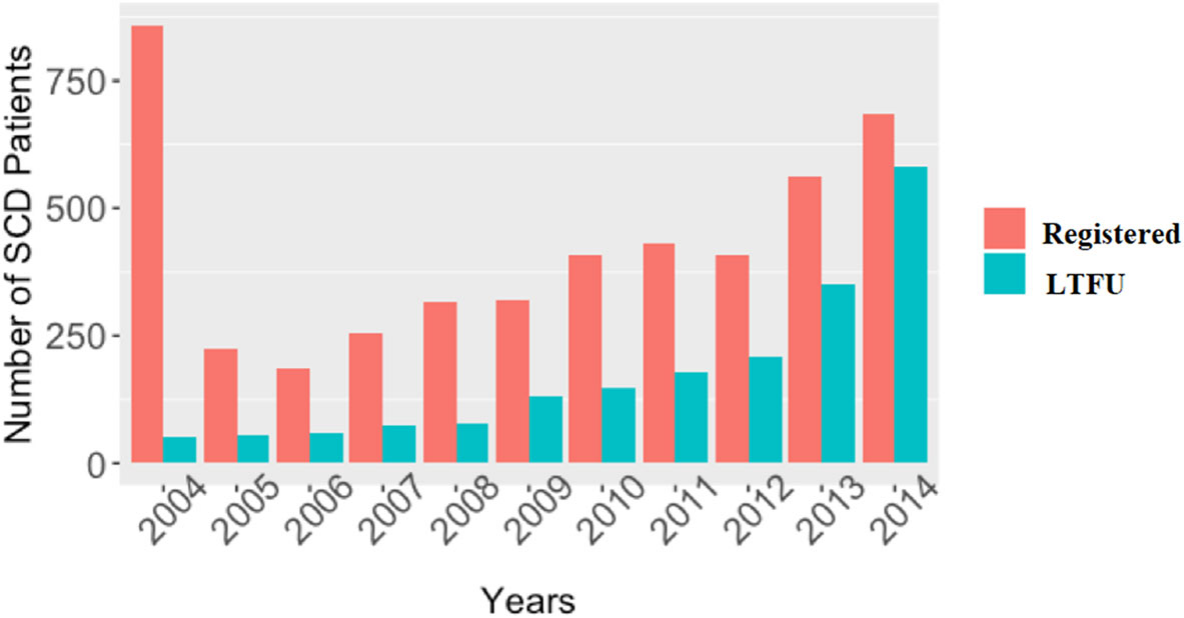
Yearly enrollment and LTFU events

The trends in enrollment and LTFU patterns from 2004 to 2014 are shown in Fig. 2. The highest numbers of registrations were achieved at the beginning of the study in 2004, while the LTFU event increases with time. The number of LTFU events (Fig. 2) in any particular year is not proportional to the number of patients registered in that year since a patient may be LTFU in a different year to the one in which they were registered. Clinic attendance up to 2014 included, 2015–2016 was excluded as additional sickle cell clinics were opened at the government referral hospitals to which SCD patients were referred. Therefore, they cannot be categorized as LTFU as they might be accessing healthcare services elsewhere. But patients register in 2015 were 645 while in 2016 were 170. Table 2 compares the clinical and laboratory data from active patients those who are not LTFU with those who were LTFU (inactive group). This table shows the median values, interquartile range and *p*-value from the T-test statistics. Based on the *p*-values obtained there are statistically significant differences between the two groups in age, mean cell volume (MCV) and mean cell hemoglobin concentration (MCHC). The median age of active patients (NOT LTFU) was 13 years compared to 11 years for inactive patients (LTFU). The level of MCV was slightly higher for those patients who were actively following their clinic appointment compared to those who were not.

**Table 2:** Comparison of laboratory and clinical parameters from active (NOT LTFU) and inactive (LTFU) patients

	Active patients (NOT LTFU)		Inactive patients (LTFU exclude those reported dead)		
	Median	IQR	Median	IQR	Test Statistics
Age	13.00	07.00–19.00	11.00	07.00–15.00	t = 5.696 (*p* < 0.001)
White blood cell (WBC) (10^3/uL)	13.11	10.69–16.60	13.65	10.90–17.19	t = −0.9953 (0.3197)
Neutrophils (%)	46.50	38.80–54.52	45.20	38.10–53.25	t = 1.9524 (*p* = 0.0509)
Hemoglobin (g/dL)	07.40	06.60–08.30	07.40	6.70–08.20	t = 0.0620 (*p* = 0.9505)
Red Blood Cells (RBC) (10^6/uL)	02.85	02.42–03.33	02.83	2.44–3.28	t = 0.1212 (*p* = 0.9036)
Mean Cell Volume (MCV) (fL)	78.58	72.00–85.20	77.40	71.50–83.80	t = 2.3742 (*p* = 0.0177)
Mean Cell Hemoglobin Concentration (MCHC)(g/dL)	33.33	31.60–34.90	33.80	32.27–35.10	t = −4.0359 (*p* < 0.001)
Red Cell Distribution Width (RDW) (%)	22.00	19.70–24.40	22.30	19.80–24.70	t = −0.2216 (*p* = 0.8247)
Platelets (10^3/uL)	417.00	304.00–522.00	409.50	312–522	t = −0.0655 (*p* = 0.9478)
Haematocrit (%)	22.22	19.70–22.20	22.80	19.60–24.50	t = 1.5341 (*p* = 0.1251)

**Fig. 3:**
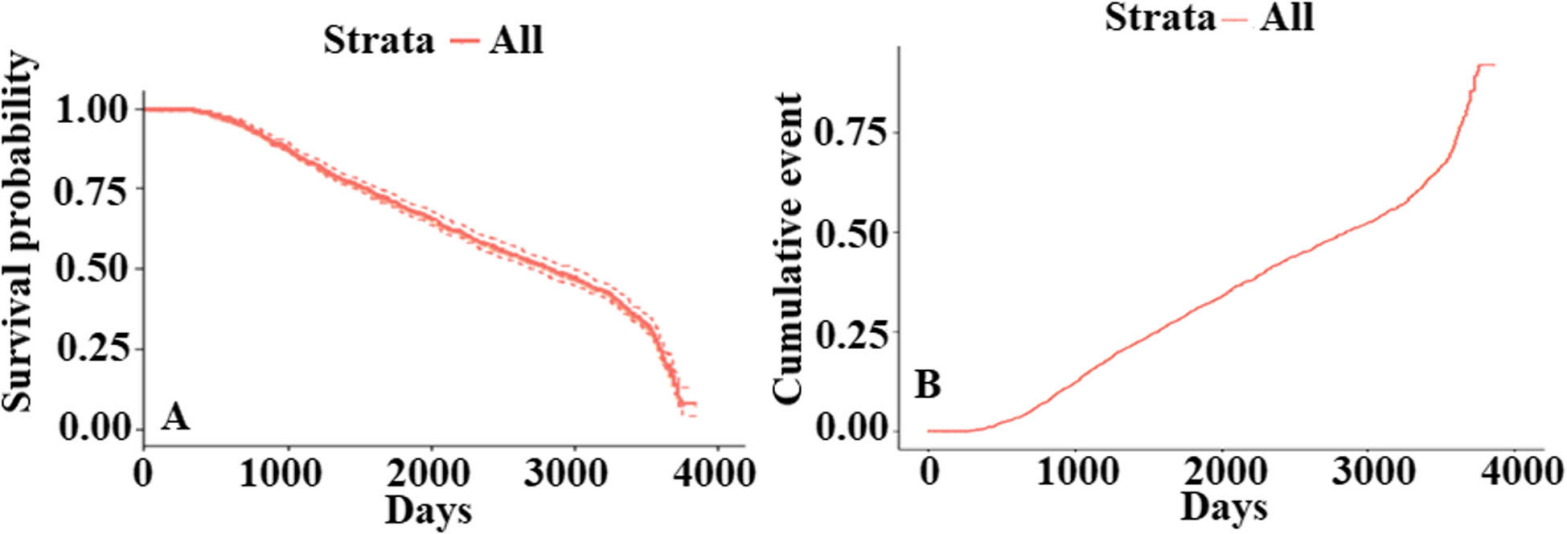
Survival probabilities and cumulative LTFU events with time in Muhimbili sickle cohort

Survival analysis is used to model survival time or the time until the event of interest. For our case, our event was LTFU. Figure 3a shows the survival probability of SCD patients over time and cumulative LTFU events over time (Fig. 3b) with a 95% confidence interval using the Kaplan-Meir method. At the beginning of the study, the survival probability was one which is expected since no LTFU events have occurred. The curve is horizontal up to around 300 days (> 9 months). The median survival time was 2848 days (7.8 years), with a 95% confidence interval of [2716–3004] days. By 2013, 10 years from the beginning of the MSC, the survival probability is around 0.2113 (21.13%), which means a retention rate of 78.87%.

**Fig. 4:**
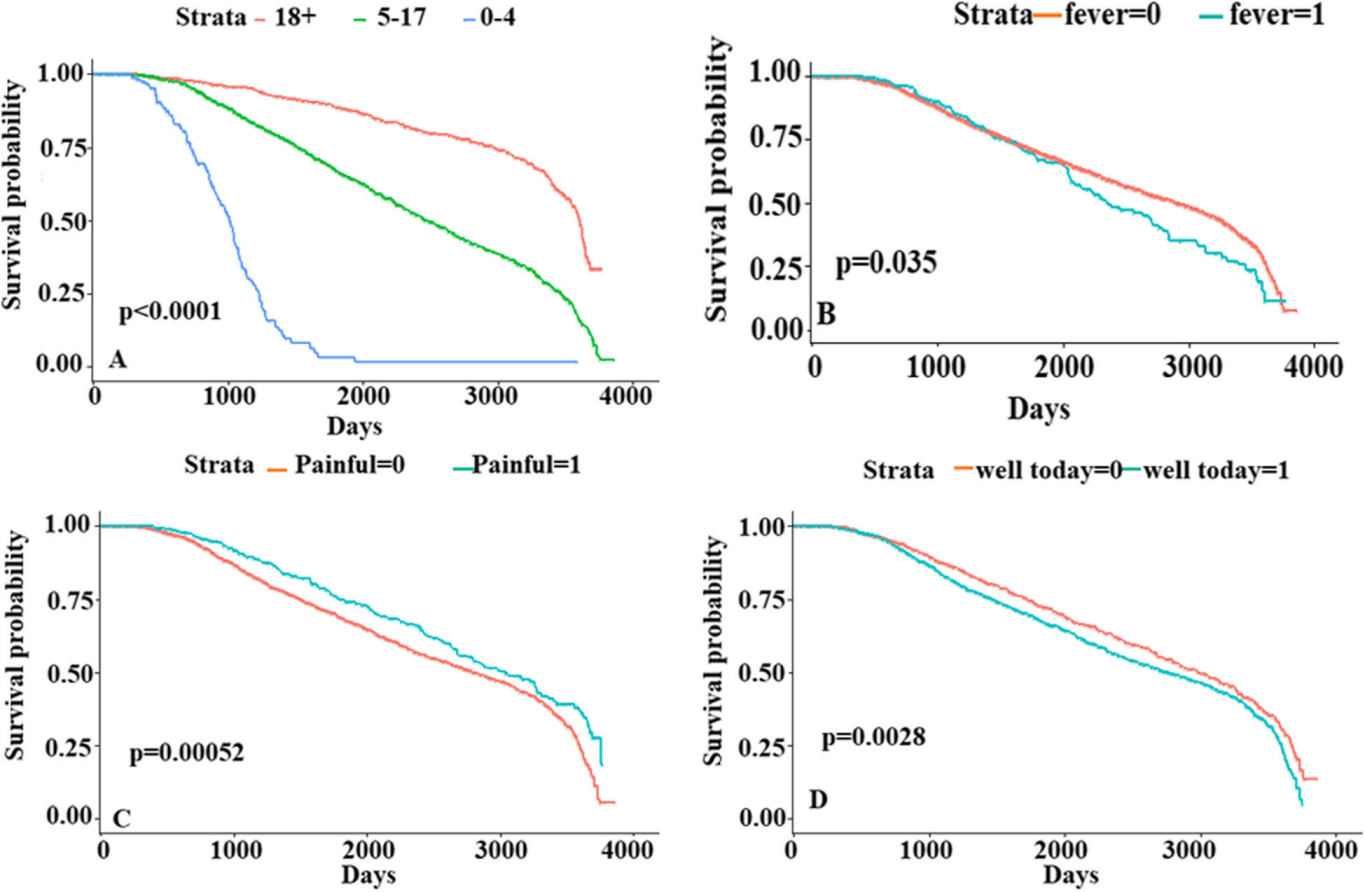
Survival probabilities by age and clinical data (fever, painful episodes and well today)

In addition to the survival trends, we compared the survival probabilities for different groups using the LogRank test and Cox hazard analysis (with a significant level of 5%). A univariate and multivariate Cox hazard analysis were implemented to select significant attributes (Table 4 in Appendix). Figure 4 shows survival curves for these four groups to the time LTFU events occurred. Figure 4a shows a comparison between three age groups categories demonstrating that patients who are 18 and over are least likely to be LTFU, followed by those who are 5–17 years old, with children under five the highest proportion of LTFU (*p* < 0.001). We also stratified patients based on fever, painful episodes and well today (the well variable (Fig. 4d) was used to record whether the patient was feeling well or not on that day). Patients who experience painful episodes (Fig. 4c) were the least likely to be LTFU compared to those with no painful episodes (*p* = 0.00052). Patients with fever (Fig. 4b) were more likely to be LTFU than those with no fever (*p* = 0.035). Table 3 shows the hazard ratios from the Cox proportional hazard model, with the age group patients who are 18 and over used as a reference group. The hazard of patients aged 5–17 years is 2.61 (95% CI: 2.2324–3.0705, *p* < 0.001) times higher than those 18 and over, while for children under five, it is 14.29 times higher (95% CI: 11.0071–18.5768, *p* < 0.001) than those 18 and over.

**Table 3:** Final results of the Cox proportional hazard model with significant attributes

Attribute	Estimate	Hazard ratio	*P*-value	95% CI
White blood cells (10^3/uL)	0.0157	1.0158	0.0074	1.0042–1.0276
Hematocrit (%)	0.0235	1.0238	0.0039	1.0076–1.0404
Well today (Yes)	0.1249	1.1331	0.0495	1.0003–1.2837
Age group (5–17)	0.9624	2.6181	< 0.001	2.2324–3.0705
Age group (< 5)	2.6602	14.2995	< 0.001	11.0071–18.5768
mch: time group (0–2050 days)	−0.1286	0.8792	< 0.001	0.8403–0.9200
mch: time group (> 2050)	0.0073	1.0073	0.8265	0.9432–1.0759
mcv: time group (0–2050 days)	0.0419	1.0428	< 0.001	1.026–1.0598
mcv: time group (> 2050)	−0.0005	0.9994	0.9629	0.9745–1.0249

In the Cox model, the painful and fever variables were removed due to lack of independence with the age group and well today attributes. Using the chi-square test, age group and painful episodes, attributes were found to depend on one another with a *p*-value of 8.889e-07 and were fever and well today (*p* = 5.96e-07) interdependent. Therefore, survival curves are shown, but only the hazard ratio result for well today. The hazard ratio of those who are well was 13.31 (95% CI: 1.0003–1.2837, *p* = 0.0495) higher than those who are not well-meaning those who felt well were more likely to be LTFU than those who were feeling unwell. The hazard ratio for patients with white blood cell (WBC) counts above average (14.34) was 1.58 (95% CI:1.0042–1.0276, *p* = 0.0074) higher than those below average, indicating those with an above-average WBC count are more likely to be LTFU than those with lower WBC. Patients with hematocrit levels above average value (22.69) have a hazard ratio of 2.38 (95% CI: 1.0076–1.0404, *p* = 0.0039) higher than those with a value below average, indicating those with above-average hematocrit values are more likely to be LTFU.

During the model, fitting mean corpuscular hemoglobin (MCH) and mean cell volume (MCV) were found to be highly significant but violating proportionality assumptions. To rectify this, the stratification of their variable was performed by splitting the follow-up period. Table 5 in the Appendix shows the results of proportionality assumptions after stratification, so all attributes are satisfying this assumption. We considered two follow-up intervals, i) 0–2050 days and ii) 2050 to the end of the study. The effect of MCV and MCH on LTFU is limited to the first period (0–2050 days). Patients with MCV value above average (77.73) have a hazard ratio of 4.28 (95% CI: 1.0260–1.0598, *p* < 0.001) higher than those below average and hence patients with MCV above average are more likely to be LTFU than those below the average value. Patients with MCH values higher than average value have a hazard ratio of 12(95% CI: 0.8403–0.9200, *p* < 0.001) less than those below the average value, meaning those with lower MCH values are less likely to be LTFU (Table 3).

## Discussion

This paper identifies patterns and factors associated with LTFU events in a large cohort of sickle cell patients receiving care at Muhimbili Hospital in Tanzania between 2004 and 2014. The proportion of patients LTFU was 35.19% (Fig. 1), with another 6.69% had died. The only mechanism for patient follow-up was a reminder of a clinic appointment by telephone. Still, our results demonstrate that telephone calls are not enough to retain all patients in the study. The increase in the number of patients who are LTFU over time shows the importance of improving or devising new methods that will improve patients’ adherence to their clinic appointments. We identified that children under 5 years were more likely to be LTFU than older age groups. This coincides with the findings by Mutanga, who reported higher incidences of LTFU in children with HIV aged five and under [13]. The author suggested dealing with stigma and child disclosure related issues early in the course of treatment and engagement with support groups to improve adherence to care.

The transition from pediatric to adult care has been identified as a phase with a high number of LTFU events among patients with chronic conditions [21]. Our study shows that patients in the age range of 5–17 years were more likely to be LTFU than those 18 and over. Reasons can include fear of leaving familiar pediatric clinics and inadequate preparation for adult care [22, 23]. In countries such as Tanzania, many children move to boarding schools at the age of 13, which may result in clinic appointments being missed and there are no strategies to handle the transition from pediatric to adult care. A transition program could be introduced that include visits by children to adults’ clinics and group meetings to discuss the transition process [23].

Patients’ health status was found to affect clinic attendance, especially in adolescent patients. A previous study looking at the barriers to regular clinic attendance in SCD patients obtained the response from the patients “why should they go to the clinic if they are well” [24]. This corresponds with our result that SCD patients who are well are more likely to be LTFU than those who were not. This highlights the need for more emphasis on clinic attendance, even when feeling well.

Hematological parameters are an effective mechanism to understand the correlation of clinical outcomes in individuals with sickle cell. There have been several studies on hematological parameters in sickle cell patients, but we went further to see if there is any relationship with LTFU patterns [25–27]. Patients whose hematocrit or MCV were above the average were more likely to be LTFU than those below average. These parameters correspond to a reduced likelihood of anemia complications and consequently feeling well [28]. A different pattern was observed with MCH, with those with a value above average were less likely to be LTFU. It is possible that those with lower MCH values were too sick to attend their clinic appointment.

Elevated white blood cell count has been associated with many complications in SCD [29]. Previous reports revealed a possible association between elevated white blood cell count and bacterial infection or vaso-occlusive crisis in sickle cell patients and elevated white blood cell count was associated with admission and frequent emergency department visits [29–31]. Our results show that those with white blood cell counts above average were more likely to be LTFU. This may suggest that they are too sick to attend appointments or they became sick before the appointment day and they were treated in other health facilities. Better follow-up of patients between clinic appointments might help address this issue and providing them with a helpline that they can call whenever they are sick instead of waiting for clinic days.

## Conclusion

We conclude that LTFU in an SCD cohort study is a problem and interventions are required. This paper shows that LTFU among sickle cell patients. The results show an association between LTFU and health, demographic or clinical variables. We recommend that cohort studies should include a protocol on how participants will be followed up to ensure they are retained in the study: this could be designing a transition program for pediatric patients to adult care, refresher training for health care workers on the effects of the LTFU and teaching them to identify patterns including clinical parameters of the patients who are more likely to be LTFU. Advocacy or patient engagement groups can provide patients with education on the importance of clinic attendance. This study is not able to determine the survival rates for those who were LTFU. Hence further investigation is needed on the fate of the patients that were LTFU.

## Data Availability

The data of this study are available from the corresponding author on reasonable request.

## Appendix

**Table 4:** Univariate and multivariate cox proportional hazard model fitting results

	Univariate Analysis			Multivariate Analysis			
	Estimate	Hazard ratio	*P*-value	Estimate	Hazard ratio	*P*-value	95% CI
Sex (Male)	0.0819	1.0853	0.1590				
Pre blood transfusion (Yes)	0.1076	1.1136	0.4840				
Pre dactylitis (Yes)	− 0.2414	0.7855	0.677				
Pre pain (Yes)	0.1135	1.1202	0.0669				
Pre chest (Yes)	0.1227	1.1305	0.415				
Pre convulsion (Yes)	−0.7094	0.4919	0.2200				
Pre leg ulceration (Yes)	−0.9425	0.3897	0.103				
Pre priapism (Yes)	0.2814	1.325	0.574				
Pain (Yes)	−0.2834	0.7532	0.0005				
Fever (Yes)	0.2295	1.2579	0.0359				
Age group (5–17)	1.0212	2.7764	<2e-16	0.9624	2.6181	< 0.0001	2.2324–3.0705
Age group (0–4)	2.9007	18.1868	<2e-16	2.6602	14.2995	< 0.0001	11.007–118.5768
Well today (Yes)	0.1839	1.2019	0.0027	0.1249	1.1331	0.0495	1.0003–1.2837
Mean corpuscular volume (fL)	−0.0049	0.9951	0.0925				
Mean cell hemoglobin (pg)	−0.0369	0.9638	3.26E-06				
Mean corpuscular hemoglobin concentration (g/dL)	−0.0812	0.922	1.43E-10				
White blood cells (10^3/uL)	0.0349	1.0355	8.74E-15	0.0157	1.0158	0.0074	1.0042–1.0276
Red blood cells (10^6/uL)	0.0421	1.043	0.28				
Mean platelet volume (fL)	−0.1372	0.8718	4.58E-06				
Hemoglobin concentration (g/dL)	−0.0371	0.9635	0.0753				
Diastolic blood pressure	−0.0047	0.9953	0.156				
Systolic blood pressure	−0.0183	0.9819	1.29E-13	−0.0078	0.9922	0.0022	0.9872–0.9972
Temperature	0.109	1.1151	0.0485				
Hematocrit (%)	0.0034	1.0034	0.618	0.0235	1.0238	0.0039	1.0076–1.0404

Variables starting with “Pre” were attributes that were collected from patients asking on any episodes that they faced since their last clinic that required OPD attendance or admission

Fever this attribute explains if one has fever (1) or does not have(0); painful this attribute says if one has painful episodes (1) or not (0) and well_today states if one is well (0) or not well

**Table 5:** Cox proportional test results on assumptions of proportionality

	rho	chisq	*p*-value
White blood cells (10^3 u/L)	−0.0007	5.78E-04	0.9808
Hematocrit (%)	0.0521	3.13E+ 00	0.0770
Well today (Yes)	−0.0431	2.16E+ 00	0.1420
Age group (5–17)	−0.0455	2.13E+ 00	0.1445
Age group (0–4)	−0.0440	2.16E+ 00	0.1420
mch:time_group(0–2050 days)	−0.0106	1.33E-01	0.7158
mch:time_group(> 2050)	0.0571	4.52E+ 00	0.0335
mcv:time_group(0–2050 days)	0.0042	2.07E-02	0.8855
mcv:time_group(> 2050)	−0.0467	3.24E+ 00	0.0719
GLOBAL	NA	1.50E+ 01	0.1312

## Abbreviation

LTFU: Loss to follow-up
SCD: Sickle Cell Disease
SCP: Sickle Cell Patient
MNH: Muhimbili National Hospital
MUHAS: Muhimbili University of Health and Allied Sciences
HU: Hydroxyurea
MCV: Mean Corpuscular Volume
MCH: Mean Cell Hemoglobin
MCHC: Mean Corpuscular Hemoglobin Concentration
WBC: White Blood Cells
RBC: Red Blood Cells
MPV: Mean Platelet Volume
HB: Hemoglobin Concentration
HR: Hazard Ratio
CI: Confidence Interval
